# Therapeutic Zfra4-10 or WWOX7-21 Peptide Induces Complex Formation of WWOX with Selective Protein Targets in Organs that Leads to Cancer Suppression and Spleen Cytotoxic Memory Z Cell Activation In Vivo

**DOI:** 10.3390/cancers12082189

**Published:** 2020-08-05

**Authors:** Wan-Pei Su, Wan-Jen Wang, Jean-Yun Chang, Pei-Chuan Ho, Tsung-Yun Liu, Kuang-Yu Wen, Hsiang-Ling Kuo, Yu-Jie Chen, Shenq-Shyang Huang, Dudekula Subhan, Yu-An Chen, Chen-Yu Lu, Chia-Yun Wu, Sing-Ru Lin, Ming-Hui Lee, Ming-Fu Chiang, Chun-I Sze, Nan-Shan Chang

**Affiliations:** 1 Laboratory of Molecular Immunology, Institute of Molecular Medicine, National Cheng Kung University, College of Medicine, Tainan 70101 Taiwan; annie1124@gmail.com (W.-P.S.); mesha19871001@gmail.com (W.-J.W.); jeanyunc@gmail.com (J.-Y.C.); peggy821124@gmail.com (P.-C.H.); T16074056@mail.ncku.edu.tw (T.-Y.L.); T16074022@mail.ncku.edu.tw (K.-Y.W.); 100712028@gms.tcu.edu.tw (H.-L.K.); shelly556119@gmail.com (Y.-J.C.); louis19862002@gmail.com (S.-S.H.); dsubhan71@gmail.com (D.S.); momofish0716@gmail.com (Y.-A.C.); jllsh99@hotmail.com (C.-Y.L.); jawork520@gmail.com (C.-Y.W.); singrulin@gmail.com (S.-R.L.); estilee@gmail.com (M.-H.L.); 2 Department of Neurosurgery, Mackay Memorial Hospital, Taipei 10449, Taiwan; chiang66@gmail.com; 3 Department of Cell Biology and Anatomy, National Cheng Kung University, Tainan 70101, Taiwan; chuni.sze@gmail.com; 4 Advanced Optoelectronic Technology Center, National Cheng Kung University, Tainan 70101, Taiwan; 5 Graduate Institute of Biomedical Sciences, College of Medicine, China Medical University, Taichung 40402, Taiwan

**Keywords:** Hyal-2^+^ CD3^-^ CD19^-^ lymphocyte, Z lymphocyte, WWOX, phosphorylation, Zfra, Hyaluronan, Hyal-2

## Abstract

Synthetic Zfra4-10 and WWOX7-21 peptides strongly suppress cancer growth in vivo. Hypothetically, Zfra4-10 binds to the membrane Hyal-2 of spleen Z cells and activates the Hyal-2/WWOX/SMAD4 signaling for cytotoxic Z cell activation to kill cancer cells. Stimulation of membrane WWOX in the signaling complex by a WWOX epitope peptide, WWOX7-21, is likely to activate the signaling. Here, mice receiving Zfra4-10 or WWOX7-21 peptide alone exhibited an increased binding of endogenous tumor suppressor WWOX with ERK, C1qBP, NF-κB, Iba1, p21, CD133, JNK1, COX2, Oct4, and GFAP in the spleen, brain, and/or lung which led to cancer suppression. However, when in combination, Zfra4-10 and WWOX7-21 reduced the binding of WWOX with target proteins and allowed tumor growth in vivo. In addition to Zfra4-10 and WWOX7-21 peptides, stimulating the membrane Hyal-2/WWOX complex with Hyal-2 antibody and sonicated hyaluronan (HAson) induced Z cell activation for killing cancer cells in vivo and in vitro. Mechanistically, Zfra4-10 binds to membrane Hyal-2, induces dephosphorylation of WWOX at pY33 and pY61, and drives Z cell activation for the anticancer response. Thus, Zfra4-10 and WWOX7-21 peptides, HAson, and the Hyal-2 antibody are of therapeutic potential for cancer suppression.

## 1. Introduction

Zinc finger-like protein that regulates apoptosis (Zfra) is a naturally occurring 31-amino-acid cytosolic protein [1,2,3,4]. We have reported that Zfra participates in tumor necrosis factor (TNF) signaling and mitochondrial apoptosis [1,2,3,4]. Transiently overexpressed Zfra physically interacts with TNF receptor adaptor proteins such as TRADD, FADD, and RIP, and thereby affects the outcome of the TNF cytotoxic pathway [1,2,3,4]. Zfra restricts the functions of nuclear factor NF-κB, JNK1 kinase, and tumor suppressors p53 and WWOX [1,2,3,4,5]. The cytosolic level of Zfra is very low and can be upregulated by stress stimuli [1,2,3,4,5]. When Zfra is synthesized in the cytoplasm, the new Zfra protein rapidly binds to cytosolic proteins and renders the degradation of the Zfra-bound proteins [6]. Notably, the degradation event is ubiquitin-/proteasome-independent [6].

Zfra is a potent agent in blocking cancer growth [1,2,3,4,6]. To determine the anticancer regions in Zfra, we synthesized Zfra peptides in a stepwise manner and then narrowed the potent anticancer segments down via in vivo experiments. We determined that synthetic full-length Zfra1-31 and truncated Zfra4-10 peptides effectively prevent the growth of many types of cancer cells in both immune-competent and immune-deficient mice [6]. In contrast, Zfra peptides are not effective in killing cancer cells in vitro. Zfra-mediated cancer suppression is associated with downregulation of WWOX with S14 phosphorylation (p14-WWOX) in cancer cell-infiltrating organs [6]. Additionally, Zfra strongly inhibits cancer metastasis, cancer stem cell development, and spontaneous cancer formation [6].

Remarkably, Zfra restores memory loss and suppresses the symptoms of Alzheimer’s disease (AD) in triple transgenic mice by reducing aggregate formation for TRAPPC6A, TIAF1, tau, and amyloid beta proteins [7]. Zfra also suppresses NF-κB-mediated inflammatory response in vivo, thereby reducing inflammation-induced neurodegeneration and neural damage [7]. Alteration of Ser8 residue for phosphorylation, i.e., S8G, abolishes Zfra polymerization and its anticancer function [6]. Whether Zfra(S8G) mitigates the AD-like symptoms in mice remains to be established. In parallel with the inhibition of cancer progression [6], Zfra suppression of AD progression correlates with inhibition of pS14-WWOX in the AD brain lesions [7].

Zfra strongly prevents cancer growth in vivo [6]. However, when ongoing solid tumors are under establishment in mice, Zfra cannot effectively suppress the tumor cell growth [6]. The failure is due, in part, to over self-polymerization of Zfra or its conjugation with blood proteins that results in loss of the anticancer efficacy in vivo [6]. The injected Zfra peptides in circulation become polymerized, exhibit self-fluorescence, and are mainly trapped or filtered in the spleen but not in other organs [6]. Zfra binds to the membrane receptor Hyal-2 in non-T/non-B spleen lymphocytes, designated Hyal-2^+^ CD3^−^ CD19^−^ or Z (for Zfra-binding) cells [6]. Gene expression profiling of naïve Z cells from T/B-deficient NOD-SCID mice has been deposited in the GEO database (Accession: GSE98409; ID: 200098409).

Here, we demonstrated that when tumor cells start to grow in vivo, non-activated or naive spleen Z cells translocate to the cancer lesions but cannot kill cancer cells. Exogenous Zfra peptides effectively induce Z cell activation in vivo or in vitro. These activated Z cells are potent in suppressing cancer cell growth in vivo [6,8]. Spleen Z cells are activated to fight against cancer by priming mice with Zfra [6,7,8]. However, T/B-deficient NOD-SCID mice are hard to prime by Zfra in vivo to fight cancer cells. Mechanisms of this regard are unknown. Activated Z cells recognize many cancer cell types, suggesting that polymerized Zfra exhibits cancer-like antigens so that activated Z cells can recognize and destruct cancer cells [6]. Zfra is not toxic and does not cause damage to organs, implying its therapeutic potential. How Z cells become activated in response to Zfra stimulation is largely unknown. The signal pathway was investigated in this study.

Z cell differentiation is different from that of T cells. Indeed, differentiation of T and Z cells is controlled, in part, by WWOX. For example, ionophore/phorbol ester-mediated forced differentiation of leukemia T cells or normal T cells requires WWOX phosphorylation at Ser14, but dephosphorylation at pY33 and pY61 [9]. The matured T cells fail to block cancer growth [9,10,11]. By comparison, we showed here that Zfra suppresses WWOX phosphorylation at S14, Y33, and Y61 to allow spleen Z cell activation.

While Z cell activation for the memory anticancer response does not require pre-exposure of Z cells to the cancer antigens [6], we tested the hypothesis that Zfra4-10 binds to membrane Hyal-2 of spleen Z cells and activates the Hyal-2/WWOX/SMAD4 signaling, which is needed for cytotoxic Z cell activation to kill cancer cells. We focused on boosting Zfra function in killing cancer cells and developing therapeutic agents in activating spleen Z cells for cancer treatment in vivo. We determined that Zfra-activated Hyal-2^+^ Z cells require the Hyal-2/WWOX/SMAD4 signaling and de-phosphorylation of WWOX at Y33 and Y61. Activated Z cells relocate to the cancer lesions to cause cancer cell death, and this correlates with S14 de-phosphorylation of WWOX in the cancer lesions.

## 2. Results

### 2.1. Zfra and WWOX Peptides Suppress Cancer Growth and Metastasis In Vivo

**Figure 1:**
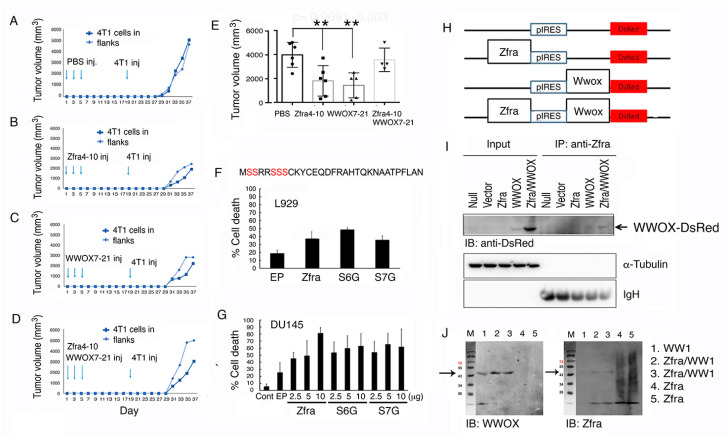
Functional neutralization between Zfra and WWOX in controlling cancer growth. (**A**–**E**) BALB/c mice received tail vein injections of an indicated synthetic peptide (2 mM in 100 μL PBS) or PBS only (100 μL) every other day for three times in a week, followed by resting for two weeks and then inoculating with mouse 4T1 breast cancer cells in both flanks. The kinetics of tumor growth in a group of individual mice is shown (A–D) and the end points of tumor volumes are shown (E). Statistical analysis: ** *p* < 0.005, Student’s t test (test samples versus PBS group). The last group (at right) is not statistically significant as versus the PBS group. The n number is shown in each bar. See Figure S1 for detailed kinetics for all mice. (**F**,**G**) Murine L929 cells or human prostate cancer DU145 cells were electroporated with an indicated Zfra cDNA expression construct (wild type, S6G or S7G mutant) and cultured for 24 h. By flow cytometry, the extent of apoptosis at SubG0/G1 phase is shown (*n* = 5). (**H**,**I**) Zfra and WWOX cDNAs were constructed in a bicistronic pIRES-based vector (H). By transient overexpression in COS7 cells, Zfra/WWOX-DsRed complex is shown in the co-immunoprecipitates (~76 kDa). The Whole Blots for Western Blot analysis for Figure 1I are shown in Figure S7. (**J**) Recombinant WW1 (~12 kDa and polymerized to 45 kDa) was mixed with Zfra peptide, incubated at room temperature for 24 h, and subjected to reducing SDS-PAGE. Zfra covalently binds WW1. Figures with digital data for Western blots 1I and 1J are shown in Figure S11.

The Zfra4-10 peptide is potent in protecting immune-deficient and -competent mice against the growth of many types of cancer cells [6,7,8]. Here, in a syngeneic model, BALB/c mice received tail vein injections of synthetic Zfra4-10 peptide (2 mM in 100 μL PBS) every other day for three times in a week, followed by resting for two weeks and then inoculating with mouse 4T1 breast cancer cells in both flanks. Zfra enabled the mice to resist the growth of 4T1 cells significantly stronger than untreated control mice (Figure 1A,B and Figure S1). WWOX7-21 peptide is a short stretch of surface-exposed epitope of WWOX and is potent in cancer suppression [9]. Under similar conditions, WWOX7-21 peptide protected mice against 4T1 cell growth (Figure 1C). In contrast, when mice received both Zfra4-10 and WWOX7-21 peptides, the anticancer effect from both peptides were totally lost (Figure 1D). The end point data for tumor growth are shown (Figure 1E).

### 2.2. Ser6 and Ser7 Are Not Involved in Zfra-Mediated Cell Death

Zfra possesses five potential phosphorylation sites at serines (Figure 1F) [2]. Alteration of Ser8 to Gly8 abolishes self-polymerization and the proapoptotic function of Zfra [2,6,7]. To determine whether Ser6 or Ser7 is necessary for Zfra to induce apoptosis, Ser6 and Ser7 were altered to Gly6 and Gly7, respectively. EGFP-tagged Zfra, Zfra (S6G), or Zfra (S7G) was transiently overexpressed in murine L929 cells or human prostate cancer DU145 cells, followed by culturing for 24 h. These cells were then harvested for cell cycle analysis by flow cytometry. All the aforementioned expression constructs induced apoptosis, as measured by determining the extent of SubG0/G1 phase (Figure 1F,G), suggesting Ser6 and Ser7 are not involved in Zfra-mediated apoptosis.

### 2.3. Binding of Zfra with the First WW Domain of WWOX Leads to Nullification of Each Other’s Function in Cancer Suppression

Newly synthesized Zfra covalently conjugates with cellular proteins [6]. Once covalently interacted with Zfra (designated as zfration), the zfrated proteins undergo rapid degradation independently of the proteasome/ubiquitination system [6,7]. Zfra binds to WWOX at both the *N*-terminal WW domain and the *C*-terminal short-chain alcohol dehydrogenase/reductase (SDR) domain and suppresses WWOX phosphorylation at Tyr33 and its apoptotic function [1,2,3,4]. To further validate the binding of Zfra with WWOX, we utilized the bicistronic pIRES-based vector to make Zfra and WWOX constructs (Figure 1H). These constructs were transiently overexpressed in COS7 cells, followed by processing co-immunoprecipitation with Zfra antiserum. In reducing SDS-PAGE, Zfra antisera precipitated a Zfra/WWOX-DsRed complex (~76 KDa) (Figure 1I), suggesting that Zfra covalently binds to WWOX. Additionally, we used the recombinant first WW domain (WW1) to mix with the Zfra peptide. The mixture was incubated at room temperature for 24 h. Under reducing SDS-PAGE, Zfra was shown to bind WW1 (Figure 1J), again indicating a covalent complex formation between WW1 and Zfra. The complex formation correlates with functional nullification between WWOX and Zfra in suppressing cancer growth (Figure 1D,E and Figure S1).

### 2.4. Increased Binding of Endogenous WWOX with Specific Proteins in Organs of Mice Correlates with Cancer Growth Suppression

**Figure 2:**
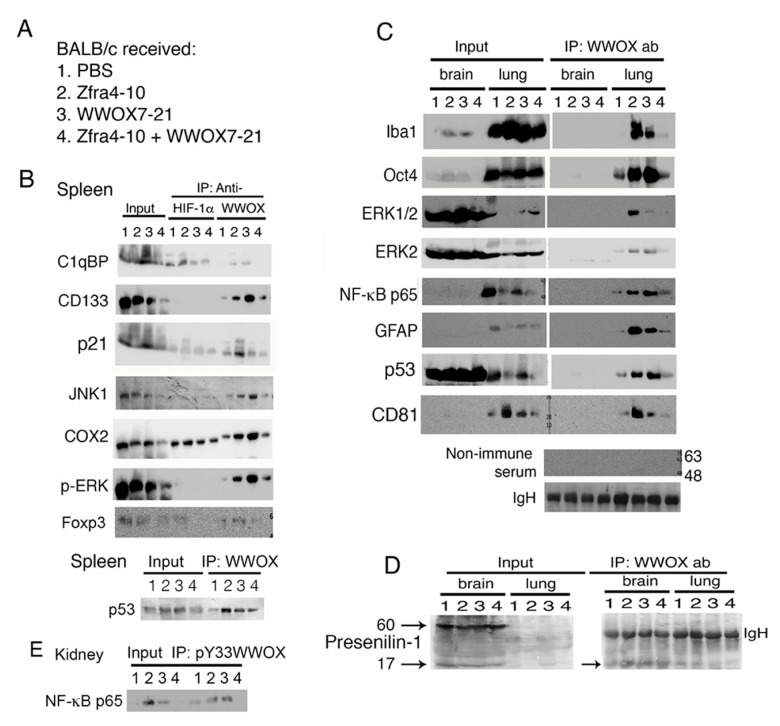
Zfra or WWOX peptide upregulates the binding of endogenous WWOX with specific proteins in organs of mice, which correlates with cancer growth suppression. (**A**) BALB/c mice received tail vein injections of Zfra4-10 and/or WWOX7-21 peptides, followed by inoculation with 4T1 breast cancer cells two weeks later and sacrifice 18 days later. (**B**) By co-immunoprecipitation, binding of HIF-1α with C1qBP and COX2 was increased by 30 to 50% in mice treated with Zfra4-10. Additionally, increased binding of WWOX with C1qBP and other indicated proteins was shown in the spleen of mice treated with either Zfra4-10 or WWOX7-21 peptide (>50 to 90%). These events correlate with cancer growth suppression. In combination of both peptides, the complex formation of WWOX and target proteins was reduced, which correlates with increased tumor growth. The Whole Blots for Western Blot analysis for Figure 2B are shown in Figure S8. (**C**) Endogenous WWOX strongly bound to Iba1, Oct4, ERK, NF-κB p65, and GFAP in the lung of mice treated with Zfra4-10 or WWOX7-21 peptide. In controls, non-immune serum was used for co-immunoprecipitation and equal loadings of protein A/G beads are reflected by IgH bands. The Whole Blots for Western Blot analysis for Figure 2C are shown in Figure S9. (**D**) Binding of WWOX with presenilin-1 was not observed in the brain of mice treated with PBS, or Zfra4-10 and/or WWOX7-21 peptides. (**E**) The binding status of pY33-WWOX with NF-κB p65 is shown in the kidney. The Whole Blots for Western Blot analysis for Figure 2D,E are shown in Figure S10. Figures with digital data for Western blots 2B to 2E are shown in Figure S12.

We investigated whether Zfra and WWOX peptides functionally counteract each other and thereby fail to suppress cancer growth in vivo (Figure 1A,D). BALB/c mice received tail vein injections of Zfra4-10 and/or WWOX7-21 peptides, followed by inoculation with syngeneic 4T1 breast cancer cells two weeks later (Figure 2A). Mice were sacrificed 18 days later. By co-immunoprecipitation, HIF-1α physically bound to C1qBP and COX2 in the spleen (Figure 2B). The binding was increased by 30 to 50% in mice treated with Zfra4-10 (Figure 2B). Mice, receiving either Zfra4-10 or WWOX7-21 peptide, had increased binding of WWOX with C1qBP, CD133, p21, JNK1, COX2, p-ERK, Foxp3, and p53 in the spleen (Figure 2B). The increased binding correlates with suppression of cancer growth (Figure 1B,C). However, in mice receiving both Zfra4-10 and WWOX7-21 peptides, WWOX had reduced binding with the target proteins down to a basal level (Figure 2B), and cancer growth was significantly increased (Figure 1A,D). Under similar conditions, endogenous WWOX strongly bound to Iba1, Oct4, ERK1/2, NF-κB p65, GFAP, and p53 in the lung of mice treated with Zfra4-10 or WWOX7-21 peptide (Figure 2C). The binding did not occur in the brain (Figure 2C). Additionally, binding of WWOX with presenilin-1 (60 kDa) did not occur in the brains of mice treated with PBS, or Zfra4-10 and/or WWOX7-21 peptides (Figure 2D). However, WWOX bound to a degradation product of presenilin-1 of 17 kDa in the brain and lung (Figure 2D). Similarly, the binding status of pY33-WWOX with NF-κB p65 is shown in the kidney, in which the binding was increased when Zfra4-10 or WWOX7-21 peptide was used (Figure 2E). The indicated proteins are involved in cell proliferation, tumor suppression, signaling, and stemness in the spleen, brain, and lung.

### 2.5. Zfra-Mediated Cancer Suppression Involves Hyal-2 and WWOX of the Hyal-2/pY33-WWOX/SMAD4 Signaling

**Figure 3:**
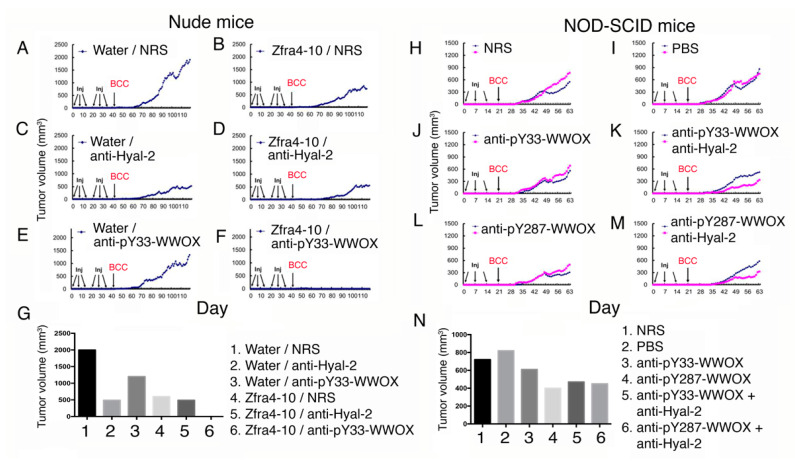
Zfra peptide, Hyal-2 antibody, or WWOX phospho-antibody inhibits basal cell carcinoma (BCC) growth in nude mice. Nude mice were pretreated with Zfra4-10 peptide (3 mM in 100 μL H2O) or sterile Milli-Q H2O via tail vein injections for three consecutive weeks, followed by injecting with an indicated antibody or normal rabbit serum (10 μL; diluted with 90 μL PBS). A week later, mice were subcutaneously inoculated with skin cancer BCC cells (two million cells on flank). A representative data is shown from two experiments. (**A**,**C**,**E**) In the control groups, sterile Milli-Q water (100 μL) and normal rabbit serum (10 μL in 100 μL H2O) did not block BCC growth (**A**). Water with Hyal-2 antiserum strongly suppressed BCC growth (**C**), whereas water with pY33-WWOX was less effective (**E**). (**B**,**D**,**F**) In the Zfra groups, Zfra4-10 peptide and normal rabbit serum suppressed BCC growth by ~75% (**B**), and Zfra4-10 peptide plus pY33-WWOX antiserum strongly blocked BCC growth by > 99% (**F**). There was no synergistic effect between Zfra4-10 peptide and anti-Hyal-2 antibody in achieving a greater suppression (~75% suppression) (**D**). (**G**) A bar graph shows an average of two tumor volumes. (**H**–**N**) NOD-SCID mice received three injections of an indicated antiserum (or normal serum or Milli-Q water) in a week and were then allowed to rest for one week followed by inoculation with BCC cancer cells. The extent of tumor growth was examined. The bar graph shows an average of two tumors.

Zfra binds to membrane Hyal-2 to activate spleen memory cytotoxic Z cells to kill cancer cells [2,6,10,11,12,13]. Additionally, when Hyal-2/pY33-WWOX/SMAD4 complex is overexpressed, hyaluronan binds to Hyal-2 and activates the signaling of Hyal-2/pY33-WWOX/SMAD4 to cause apoptosis in vivo [2,6,10,11,12,13]. To establish the role of Hyal-2 and WWOX in cancer suppression, nude mice received tail vein injections with sterile Milli-Q water or Zfra4-10 peptide (1 mM in 100 μL sterile Milli-Q water) in every two other days for three times. One week later, the mice received normal rabbit serum (NRS) or a specific antiserum (10 μL serum plus 90 μL PBS) for another three consecutive injections (Figure 3A–F). The growth of skin basal cell carcinoma (BCC) tumor was blocked in mice receiving Zfra peptide or Hyal-2 antiserum (>75% inhibition; Figure 3B,C). pY33-WWOX antibody was less effective in blocking BCC growth than Hyal-2 antibody (Figure 3C,E). In controls, sterile water did not block BCC growth (Figure 3A). When mice received Zfra4-10 peptide and Hyal-2 antiserum, the extent of BCC growth was similar to that of Hyal-2 antiserum alone (Figure 3D). Notably, Zfra4-10 peptide and pY33-WWOX antiserum, in combination, completely blocked the growth of BCC (Figure 3F). The extent of tumor growth is tabulated in a bar graph (Figure 3G). Zfra is known to suppress WWOX phosphorylation at Tyr33 [2,3]. Under similar experimental conditions, antiserum against WWOX at pY33 or pY287, along with Hyal-2 antiserum, reduced the BCC tumor growth in T/B-deficient NOD-SCID mice (30 to 50% reduction; Figure 3H–N), suggesting that Zfra inhibits WWOX phosphorylation at Tyr33, which is needed for suppressing cancer growth.

### 2.6. Transfer of Purified Activated Z Cells to Recipient Mice Confers Resistance to Tumor Growth

**Figure 4:**
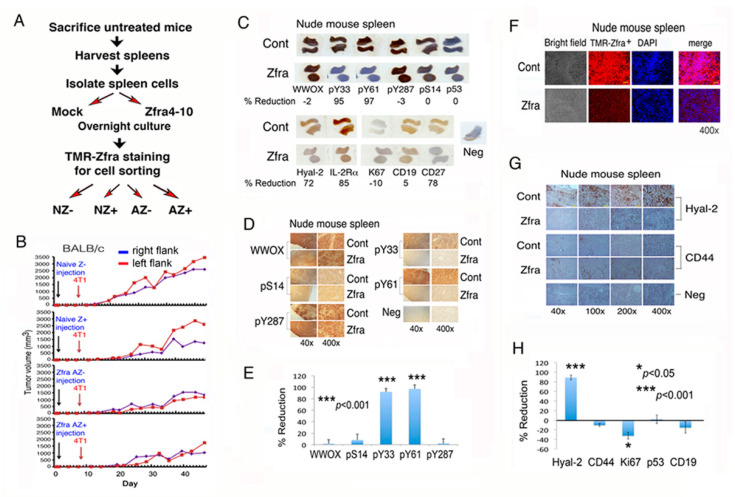
Zfra peptide suppresses WWOX phosphorylation at Y33 and Y61 that leads to Z cell expansion in the spleen. (**A**,**B**) Spleen cells were isolated from eight naïve BALB/c mice, followed by treating with or without 20 μM Zfra4-10 for 24 h in cell culture and then processing purification of TMR-Zfra+ cells using a cell sorter. The resulting naïve TMR-Zfra- (NZ-), naïve TMR-Zfra+ (NZ+), Zfra-activated TMR-Zfra- (AZ-), and Zfra-activated TMR-Zfra+ (AZ+) were isolated and injected to each indicated recipient naïve nude mouse via its tail vein. One week later, breast 4T1 cancer cells were inoculated into both flanks for measuring tumor growth with time. (**C**–**E**) Nude mice received Zfra peptide (2 mM in PBS) or PBS only via tail vein injections, followed by inoculating B16F10 cells in both flanks one week later and mice sacrificed a month later. Zfra suppressed WWOX phosphorylation at Y33 and Y61 (> 95%), and inhibited the level of CD27^+^ B cells by 78% in the spleen (whole mount scans). Statistical analysis for E: *** *p* < 0.001, *n* = 5, Student’s t test (all groups versus pS14 group). (**F**–**H**) Z cell levels were low in the spleen of mice post treatment with Zfra for two months, as the spleen cells had reduced expression of Hyal-2 and Zfra. Statistical analysis for H: * *p* < 0.05, *** *p* < 0.001, *n* = 5, Student’s t test (all groups versus CD44 group).

We reported the purification of Zfra-activated Hyal-2^+^ Z cells by cell sorting and showed their suppression cancer growth in vivo [6]. Here, we isolated spleen cells from eight naïve BALB/c mice for in vitro activation. These naive spleen cells were cultured for 24 h in the presence or absence of 20 μM Zfra4-10. By cell sorting using a red-fluorescent TMR (tetramethylrodamine)-Zfra as a probe (Figure S2) [6,7], we isolated four populations of cells, including naïve TMR-Zfra– (NZ–), naïve TMR-Zfra+ (NZ+), Zfra-activated TMR-Zfra– (AZ–), and Zfra-activated TMR-Zfra+ (AZ+) cells (Figure 4A,B). These cells were then transferred to naïve BALB/c mice via tail veins. One week later, the mice were inoculated subcutaneously in both flanks with 4T1 cells. Mice harboring Zfra-activated Z cells resisted the growth of 4T1 cells (Figure 4B).

### 2.7. Zfra Causes WWOX de-Phosphorylation at Y33 and Y61 to Drive Z Cell Activation in the Spleen

Next, we examined the status of WWOX phosphorylation in the spleen and whether WWOX de-phosphorylation at Y33 and Y61 contributes to Z cell activation. Calcium ionophore A13827 and phorbol myristate acetate forcefully induce the maturation of leukemia T cells [9,11]. This involves de-phosphorylation of WWOX at Y33 and Y61, but increased phosphorylation at S14, in MOLT-4 T cells in five minutes or less in vitro [9]. Zfra activates Z cells probably via the membrane Hyal-2/WWOX/SMAD4 signaling [12,13,14,15,16,17]. Zfra binds to the membrane Hyal-2 as a receptor in spleen Z cells [6]. Additionally, Zfra binds WWOX7-21 in front of the first WW domain and the *C*-terminal SDR domain, as determined by co-immunoprecipitation (Figure 1I,J), and yeast two hybrid analysis [2].

Nude mice received the full-length Zfra1-31 peptide via tail vein injections once per week for three consecutive weeks, followed by resting for one week and then inoculating with B16F10 cells in both flanks. A month later, mice were sacrificed. In agreement with our previous observations [6], Zfra suppressed B16F10 growth by 78 ± 8% (*n* = 5) in nude mice, as determined by measuring the tumor volumes. At the end point of tumor growth experiments, Zfra significantly suppressed the expression of Hyal-2 and phosphorylation of WWOX at Y33 and Y61 (>90%), but no significant changes were observed for WWOX phosphorylation at S14 and Y287 in the spleen of the sacrificed mice (<5%; Figure 4C–E).

Clonal expansion of Z cells was shown in the spleen of Zfra4-10-treated nude mice within a week (Figure S3). During a prolonged treatment of nude mice with Zfra for two months, downregulation of Hyal-2, and reduction in TMR-Zfra-positive Z cells occurred in the spleen (Figure 4F–H), suggesting that Z cells relocate to a tumor-growing organ. Zfra suppressed the expression interleukin 2 receptor alpha (IL-2Rα or CD25) in the spleen (Figure 4C). IL-2Rα is overexpressed in the surface of hematological tumor cells, and is associated with the oncogenic signaling of leukemic stem cells [18,19]. Cell proliferation antigen Ki67, but not p53, was significantly increased in the spleen of Zfra-treated mice (Figure 4C–H).

In the mouse spleen, Zfra marginally increased naïve CD19^+^ B cells by less than 10% (Figure 4C–H), whereas Zfra significantly suppressed memory CD27^+^ B cells in the spleen by 78% (Figure 4C). To examine the effect of B cells, nude mice received CD19 or CD27 antibody (or PBS only) and then Zfra4-10 peptide via tail vein injections. CD19 or CD27 antibody had a marginal effect in the Zfra-mediated cancer suppression (Figure S4), suggesting that CD19^+^ or CD27^+^ B cells are not involved in Zfra-mediated cancer suppression. In this study, Zfra was resuspended in water, and its anticancer activity was reduced due to insufficient self-polymerization [6].

Together with post-tumor inoculation in mice for one month, Zfra inhibited the expression of pY33- and pY61-WWOX, Hyal-2, IL-2Rα, and CD27 in the spleen, but had no effect on CD19. Reduced expression of Hyal-2 and Zfra in the spleen suggests that activated memory Z cells participate in cancer suppression by relocating to the cancer lesions. Both CD19 and CD27 are markers of B cells. Naïve B cells have a phenotype of CD19^+^ CD27^-^, whereas memory B cells are CD19^+^ CD27^+^ [20,21,22]. Our data failed to support the involvement of B cells in assisting Z cells in fighting against cancer.

### 2.8. Zfra-Activated Z Cells Relocate to Cancer Lesions to Block Cancer Growth

**Figure 5:**
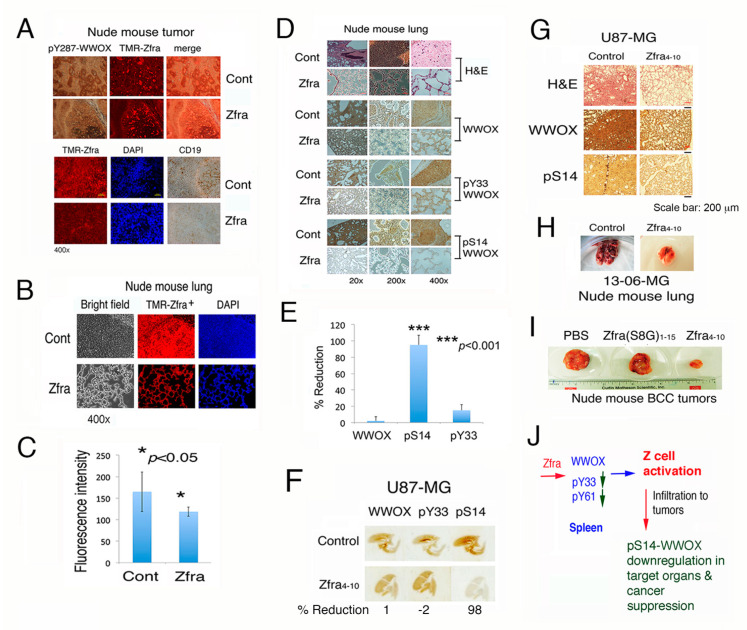
Relocation of Z cells to cancer lesions and Zfra suppression of pS14-WWOX correlates with cancer growth inhibition. (**A**) Nude mice were treated with or without Zfra once per week for three consecutive weeks, followed by inoculation with B16F10 cells. One month later, mice were sacrificed. Relocation of naïve and activated Z cells from the spleen to the tumor lesions via lymphatic vessels is shown (see red punctate). (**B**,**C**) Dramatic suppression of B16F10 melanoma growth is shown in the lung of Zfra-treated mice. As the lung turns normal, the level of Z cells is significantly reduced in the lung of Zfra-treated mice (*n* = 6). Non-activated Z cells accumulated in the B16F10 lesions of the lung but failed to kill cancer cells. Statistical analysis for C: * *p* < 0.05, n = 5, Student’s t test (Zfra group versus control group). (**D**,**E**) Suppression of cancer growth in the lung by Zfra correlates with inhibition of WWOX phosphorylation at Ser14. Statistical analysis for E: *** *p* < 0.001, n = 5, Student’s t test (all groups versus WWOX group). (**F**) Nude mice received Zfra4-10 (4 mM in 100 μL sterile MilliQ water) or an equal volume of sterile water for three consecutive weeks. A week later, these mice were inoculated with U87-MG cells on two subcutaneous sties of both flanks (2 × 10^5^ cells in 100 μL PBS). The whole mount lung tissue sections were stained with specific antibodies by immunohistochemistry and imaged by a scanner. (**G**) Compared to control mice, Zfra4-10 suppressed WWOX phosphorylation at S14 by greater than 70–90% in the lung, blocked the U87-MG tumor formation by ~50% in the skin, and inhibited U87-MG cell metastasis to the lung. Scale bar, 200 μm. (**H**) Zfra-treated nude mice resisted metastasis of glioblastoma 13-06-MG to the lung. (**I**) Compared to the Zfra4-10 peptide, Zfra(S8G)1-15 peptide failed to block BCC growth in mice. (**J**) Summary of Zfra-mediated Z cell activation in the spleen, Zfra suppression of pS14-WWOX in the cancer lesions, and Z cell killing of cancer cells in a target organ.

In response to tumor growth (Figure 4), both naïve and activated Z cells relocated from the spleen to the tumor lesions via lymphatic vessels, as determined using red fluorescent TMR-Zfra peptide. TMR-Zfra binds to the Z cell membrane Hyal-2 (Figure 5A) [6]. Compared to control mice, reduced levels (>50%) of CD19^+^ B cells were shown in the cancer lesions (Figure 5A). Expression of pY287-WWOX is shown in the tumors in both control and Zfra-treated mice (Figure 5A). pY287-WWOX is subjected to degradation by the ubiquitin/proteasome system [9,11,12,13]. In control mice, naïve Z cells infiltrated in the lung, but did not suppress the growth of metastatic B16F10 cells (Figure 5B,C). Failure of these Z cells in suppressing cancer growth is simply because they are not pre-activated by Zfra [6,7,8].

Zfra suppressed B16F10 metastasis to the lung, which correlates with a significant reduction of S14 phosphorylation of WWOX in the lung (Figure 5B–E). The observation is in agreement with our previous reports [6,7]. The overall levels of WWOX in the lung remained largely unchanged between control and Zfra-treated mice (Figure 5B–E). Y33 phosphorylation in WWOX was not affected in the lung (Figure 5B–E).

### 2.9. Zfra Suppresses pS14-WWOX Expression in the Lung and Thereby Prevents Glioblastoma Cell Metastasis to the Lung

In parallel with the aforementioned observations, we examined the effect of Zfra on the inhibition of metastasis of glioblastoma cells in vivo. Zfra inhibits the growth of certain but not all glioblastoma cells in vivo [6]. Nude mice were pre-injected with Zfra4-10 (4 mM in 100 μL sterile MilliQ water) or an equal volume of sterile water for three consecutive weeks. A week later, these mice were inoculated with glioblastoma U87-MG cells on two subcutaneous sties of both flanks (2 × 10^5^ cells in 100 μL PBS). Zfra blocked the metastasis of U87-MG cells to the lung, and this correlates with inhibition of WWOX phosphorylation at S14 but not at Y33 (Figure 5F,G). We have previously shown that Zfra-mediated suppression of pS14-WWOX leads to cancer growth suppression [6,7] and blocking of the progression of Alzheimer’s disease [7]. Under similar experimental conditions, Zfra-treated nude mice resisted the metastasis of glioblastoma 13-06-MG cells to the lung, compared to PBS controls (Figure 5H). Additionally, Zfra-treated nude mice resisted the growth of BCC cells, compared to PBS controls and Zfra(S8G) mutant peptide (Figure 5I). S8 is a phosphorylation site and is responsible for self-polymerization [6,7]. Alteration of S8 to G8 abolishes the anticancer function of Zfra [6].

Together, Zfra causes Z cell activation in the spleen and suppresses pS14-WWOX in the cancer lesions (Figure 5J). Reduction in WWOX phosphorylation at Ser14 in the cancer lesions correlates with cancer suppression. Although circulating polymerized Zfra is mainly deposited in the spleen, we propose that a small population of Zfra may go directly to the cancer lesions to suppress pS14-WWOX, thereby reducing cancer growth. Alternatively, Z cells may secrete phosphatases to reduce S14 phosphorylation in the cancer lesions or caner-infiltrated organs.

### 2.10. Sonicated Hyaluronan (HAson) Suppresses Cancer Growth In Vivo via Hyal-2/WWOX Signaling

**Figure 6:**
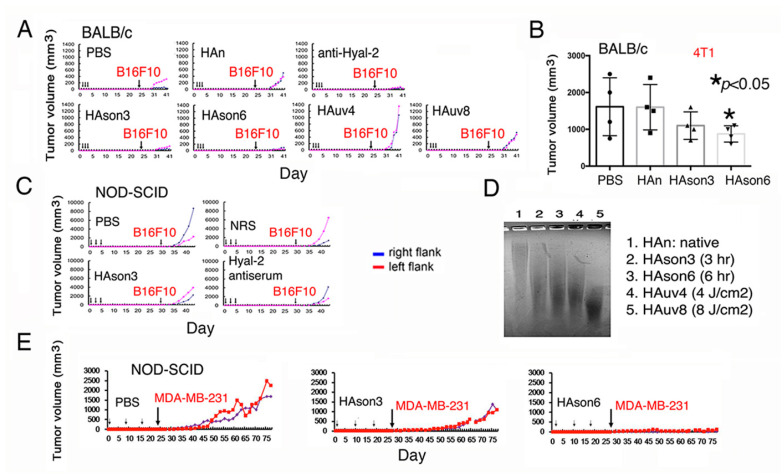
Sonicated hyaluronan (HAson) blocks melanoma and breast cancer growth in mice. (**A**) BALB/c mice pretreated with antibody against Hyal-2 (10 μL serum in 90 μL PBS), 200 μg native HA (HAn in 100 μL PBS), or 200 μg modified HA (sonicated HAson or UV irradiated HAuv) via tail vein injections for three consecutive days. 20 days later, mice were subcutaneously inoculated with B16F10 cells. (**B**) Under similar conditions, BALB/c mice received tail vein injections with hyaluronan preparations for three consecutive days, followed by inoculation with syngeneic 4T1 breast cancer cells. Statistical analysis for B: * *p* < 0.05, n = 4, Student’s t test (all groups versus PBS group). (**C**) Similarly, both Hyal-2 antibody and HAson suppressed B16F10 growth in NOD-SCID mice. (**D**) Both sonication and UV irradiation caused HA degradation, as compared to the native HA. (**E**) Similarly, HAson6 blocked breast MDA-MB-231 cell growth in NOD-SCID mice. HAn = native HA; HAson3 or 6 = HAn sonicated for three and six hours, respectively. NRS = normal rabbit serum.

Hyal-2 antibody blocked BCC growth in mice (Figure 3C). To further validate the Hyal-2/WWOX signaling in cancer suppression, BALB/c mice were pretreated with Hyal-2 IgG, medical grade hyaluronan (HAn), sonicated HA (HAson3 or HAson6 for sonication for three or six hours), and UV-irradiated HA (HAuv for four or eight hours), respectively, via tail vein injections once per day for three days. 20 days later, mice received subcutaneous inoculation with B16F10 cells. HAson3 and HAson6 effectively blocked the B16F10 growth (Figure 6A). Under similar conditions, BALB/c mice received tail vein injections with hyaluronan preparations for three consecutive days, followed by inoculation with syngeneic 4T1 breast cancer cells. HAson6 significantly suppressed the growth of 4T1 cells (Figure 6B). When NOD-SCID mice were treated similarly with Hyal-2 IgG or HAson, B16F10 growth was suppressed (Figure 6C). When subjected to sonication or UV irradiation, HA underwent partial degradation as compared to the native or untreated HA (Figure 6D). Finally, NOD-SCID mice received sonicated HAson3 or HAson6 for three consecutive weeks, followed by subcutaneous inoculation with breast MDA-MB-231 cells in both flanks. HAson6 effectively blocked the tumor growth (Figure 6E). Taken together, sonicated HA and specific antibodies against Hyal-2 act as agonists in stimulating membrane Hyal-2 in Z cells, so as to suppress cancer growth.

### 2.11. Activated Spleen Z Cells Aggressively Attack and Cause Breast Cancer Cell Death In Vitro

**Figure 7:**
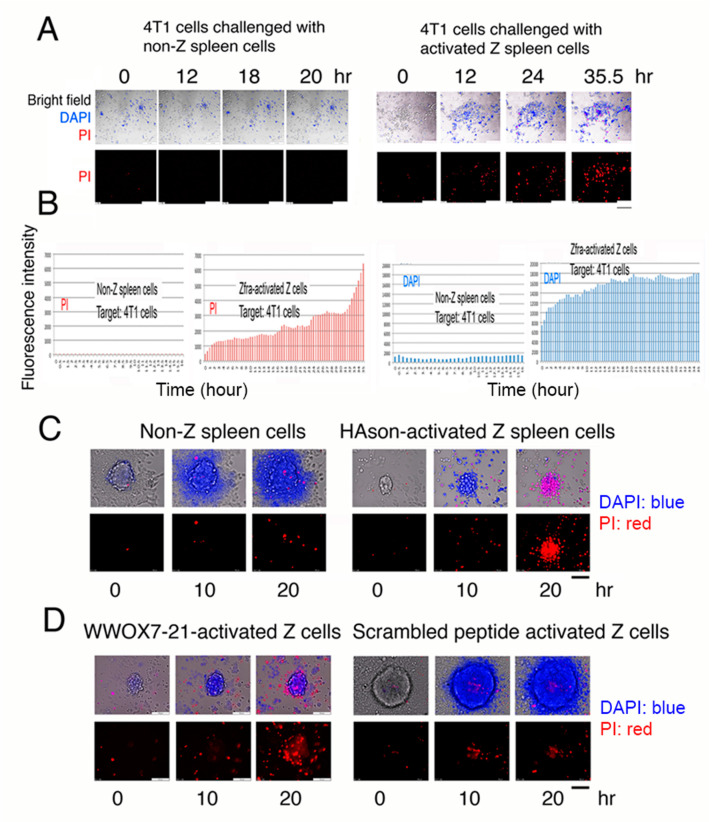
Activated Z cells attack and kill cancer cell in vitro. (**A**) Activated Z cells (8 × 10^5^ cells) purified from *Wwox* wild type B6 mice were co-cultured with mouse breast 4T1 cells for time lapse microscopy. DAPI and PI were included in the coculture. Scale bars: 100 μm (bottom right in all pictures and a black bar in bottom right). Also, see Videos S1–S3. (**B**) Activated Z cells induced 4T1 cells to rapidly pick up DAPI, followed by PI as the cells died. Non-Z spleen cells did not kill 4T1 cells. No DAPI and PI pickup occurred. Additionally, see Videos S4–S6. (**C**) Purified activated Z cells were from HAson8-treated B6 mice. Z cells killed 4T1 cells in the spheres, whereas non-Z cells had no effect. (**D**) Similarly, WWOX7-21-activated spleen Z cells killed 4T1 cells in spheres, whereas scrambled peptide had no effect. Additionally, see Videos S11–S14. Scale bars for C and D: 100 μm (at D left panel and a black bar in bottom right).

In our recent study, we have demonstrated that ceritinib, an anaplastic lymphoma kinase (ALK)-positive inhibitor for lung cancer treatment [23,24], is able induce breast cancer stem cell sphere explosion and death [10]. Whether activated Z cells attacked and killed cancer cells and cancer stem cell spheres in vitro was investigated. Naïve wild type Wwox B6 mice were treated with Zfra (1 mM in 100 μL PBS) once via tail vein injection, followed by resting for one week, and then were subjected to purification of activated Z cells in the spleen [6,7]. Activated Z cells (8 × 10^5^ cells) were co-cultured with mouse breast 4T1 cell monolayers, in the presence of nuclear stains 4’,6-diamidino-2-phenylindole (DAPI; blue fluorescence) and propidium iodide (PI; red fluorescence), for imaging by time-lapse microscopy [9,10,13,16,25]. Activated spleen Z cells underwent clonal expansion and aggressively attacked and killed 4T1 cells (Figure 7A,B; Videos S1–S3). 4T1 cells exhibited rapidly altered nuclear membrane permeability as they picked up the DAPI stain (Figure 7B). When 4T1 cells started to die, they picked up the PI stain (Figure 7B). Non-Z spleen cells failed to kill 4T1 cells (Figure 7A,B; Videos S4–S6). In a similar experiment, activated Z cells were isolated from heterozygous Wwox+/− mice and attacked 4T1 cells (Figures S5 and S6; Videos S7–S10). The 4T1 cells died in a manner with soma explosion (Videos S7–10), which is similar to our recently discovered bubbling cell death [13,16,26,27]. Next, we examined whether purified activated Z cells kill cancer stem cell spheres. Activated Z cells were isolated from HAson8-treated B6 mice and shown to kill 4T1 cells in the spheres; however, non-Z cells had no effect (Figure 7C). Under similar experimental conditions, WWOX7-21-activated spleen Z cells were purified and shown to kill 4T1 cells in spheres (Figure 7D). In controls, scrambled peptide-activated Z cells failed to kill 4T1 cells (Figure 7D). 

## 3. Discussion

We investigated whether synthetic Zfra4-10 or WWOX7-21 peptide-mediated cancer suppression occurs via the Hyal-2/WWOX/SMAD4 signaling for cytotoxic memory Z cell activation in the spleen. Both peptides are potent in suppressing cancer growth in vivo [6,9]. When mice received Zfra4-10 or WWOX7-21 peptide alone followed by 4T1 breast cancer inoculation, these mice developed endogenous complex formation of WWOX with many target proteins, including ERK, C1qBP, NF-κB, Iba1, p21, CD133, JNK1, COX2, Oct4, and GFAP in the spleen, brain, and lung. The binding event contributes to the inhibition of cancer growth in vivo. Indeed, WWOX physically binds to many cellular proteins, such as p53, Hyal-2, Smad3 and 4, JNK1, Tau, Zfra, and many others [11,12,13,14,15,26,27,28,29,30,31,32,33,34,35]. In stark contrast, when both Zfra4-10 and WWOX7-21 peptides are introduced simultaneously to mice via tail veins, these mice have significantly reduced levels of WWOX complex formation with proteins in the aforementioned organs. These mice lose their ability to resist cancer growth. Zfra4-10 binds to membrane Hyal-2 [6], and WWOX7-21 may interact with membrane WWOX (unpublished). This raises the strong scenario that both peptides drive a common signal pathway, which is Hyal-2/WWOX/SMAD4, to limit cancer growth. Overall, WWOX controls an army of proteins to maintain normal cellular physiology. Failure in this regulatory control may lead to cancer initiation and progression [36].

HIF-1α is proinflammatory and pro-survival for cancer [37]. For example, WWOX regulates the function of HIF-1α in promoting cancer progression [38]. Transmembrane protein 207 (TMEM207) plays a role in invasiveness of gastric signet ring cell carcinoma [39]. TMEM207 abolishes the binding of WWOX with HIF-1α to enhance cancer growth [39]. We determined for the first time that HIF-1α binds to C1qBP, COX2, and slightly with p21 in the spleen. Functional significance of this binding is unknown. Both C1qBP and COX2 are involved in cancer growth, progression, and inflammation [40,41]. p21 is an inhibitor of the cell cycle progression and is able to arrest the cell cycle at the G1/S and G2/M phases, whereas p21 becomes an oncogenic factor in a p53-deficient environment [42,43]. Zfra4-10 peptide increases the binding of HIF-1α with C1qBP and COX2. Additionally, Zfra4-10 or WWOX7-21 peptide increases the complex formation of WWOX with C1qBP, CD133, p21, JNK1 [44], COX2, and p-ERK [10] in the spleen, which correlates with cancer suppression in vivo. Loss of the binding between WWOX and target proteins leads to increased cancer cell growth. The complex formation of WWOX with C1qBP, CD133, p21, and COX2 in the spleen is identified for the first time.

Similarly, Zfra4-10 or WWOX7-21 peptide enhances the binding of WWOX with Iba1, GFAP, Oct4, ERK1/2, p53, and NF-κB p65 in the lungs of BALB/c mice. However, the binding is barely detectable in the brain. Iba1 (or AIF1) and GFAP are markers of brain microglial cells and astrocytes, respectively [45,46]. Their presence in the lung and skin is due to secretion of Iba1/AIF1 by inflammatory macrophages [47,48]. Whether cancer-mediated inflammatory reaction allows for relocation of brain microglia and astrocytes to the lung is being determined in our laboratory. The lung–brain axis allows a connection of lipopolysaccharide-dependent inflammation [47]. WWOX physically binds to p53, and both may induce apoptosis synergistically in vitro [5,11,14]. The peptide-induced WWOX binding with p53 and NF-κB p65 causes cancer suppression. However, dual peptides reduce the formation of WWOX/53 and WWOX/NF-κB p65 complexes and the event allows cancer growth. Our recent study showed that p53 and WWOX may functionally counteract with each other in vivo and thereby enhances cancer growth and neurodegeneration [49]. We believe that the functional antagonism between p53 and WWOX is due to reduced physical binding.

WWOX utilizes its first WW domain at the *N*-terminus to bind PPXY motif-containing proteins [35,50]. When Tyr33 is phosphorylated, WWOX has an expanded binding capability. In addition to the first WW domain, the *C*-terminal SDR domain also binds to many proteins. Although the functional significance of the binding is unknown, an enhanced binding of WWOX with its partners is critical in blocking cancer cell growth in vivo.

Supporting evidence shows that Zfra-induced Hyal-2/WWOX/SMAD4 signaling is responsible for Z cell activation and the anticancer response [6,12,13,15]. Zfra4-10 binds to membrane Hyal-2, then induces dephosphorylation of WWOX at pY33 and pY61, and finally drives Z cell activation for the anticancer response. We showed in this report and previous studies that Zfra physically binds to WWOX and many other proteins, known as zfration [1,2,3,4,6]. These complexes are resistant to dissociation by β-mercaptoethanol under reducing SDS-PAGE, suggesting that Zfra covalently binds to WWOX and other protein targets, leading to rapid degradation [6]. The degradation is ubiquitin- and proteasome-independent. Whether Ser8 in Zfra participates in the covalent binding is being determined. Based upon the Hyal-2/WWOX/SMAD4 signaling, our therapeutic agents are developed to target membrane Hyal-2 and WWOX. These agents include Zfra4-10 peptide, WWOX7-21 peptide, Hyal-2 antibody, pY33-WWOX antibody, and HAson, which are potent in inhibiting the growth of breast cancer cells and melanoma in vivo. Additionally, these agents can be utilized to induce Z cell activation in vitro to kill cancer cells in vivo.

Previously, we have shown the strong potency of full-length peptide in preventing and suppressing the growth of many types of cancer cells in both immune competent and deficient mice [6]. One of the crucial features of Zfra peptides, Zfra1-31 and Zfra4-10, is their capability in blocking cancer metastasis [6] and mitigating the progression of Alzheimer’s disease [7]. The anticancer activity of Zfra is most likely due to Zfra self-polymerization via Ser8 in vivo. When Ser8 is altered to Gly8, the anticancer function of Zfra is abolished [6]. Conceivably, over self-polymerization of Zfra caused by PBS leads to its functional inactivation, simply because the overly polymerized materials fail to activate Z cells.

We investigated the Hyal-2/WWOX/SMAD4 signaling for spleen Z cell activation. Zfra binds membrane receptor Hyal-2 to allow Z cell activation [6]. We demonstrated that activated Z cells undergo clonal expansion and rapidly attack cancer cells in vitro. Z cell is not T, B, monocyte/macrophage, or NK. We have validated the observation by gene expression profiling approaches using naïve Z cells from T/B-deficient NOD-SCID mice (NCBI GEO #GSE98409). In the Hyal-2-dependent signaling, sonicated hyaluronan (HAson) and specific antibody against Hyal-2 or pY216-Hyal-2 induce a strong anticancer response, which correlates with the occurrence of Z cell activation and the memory anticancer response in vivo [6]. How the memory anticancer response occurs in Z cells is intriguing and remains to be elucidated.

The reason for using sonication is that sonication causes hyaluronan to break down into small molecular sizes and the newly generated molecules are likely to have disordered conformation with potential covalent binding among the short HA chains. Sonication induces heat and high temperature up to 75 °C, which may induce covalent bonding due to oxidation. UV irradiation-treated HA also results in breakdown of HA long chains, which are smaller than that of sonication treatment. Nonetheless, UV-generated HA fragments are not effective in blocking cancer growth, suggesting that the Hyal-2/WWOX pathway is not activated in Z cells. Whether there is a size-dependent cancer suppression of HA is unknown. Indeed, high-molecular-weight native HA promotes cancer growth, suggesting that its binding and the agonizing effect for membrane Hyal-2 are probably not strong enough. Under physiologic conditions, membrane Hyal-2 binds the newly synthesized high-molecular-weight hyaluronan for digestion and release to the extracellular matrix and the circulation [51].

Forced T leukemia cell differentiation by calcium ionophore and phorbol ester requires rapid de-phosphorylation of WWOX at Y33 and Y287 but increased phosphorylation at S14 in less than five minutes [10,11]. This also occurs in normal T cell differentiation (Chang et al., unpublished)—that is, pS14-WWOX plays a critical role in the T cell terminal differentiation. Utilization of inducible engineered T cell receptor in precursor T cells for blocking leukemia T cell proliferation has been documented [52]. Many reports have utilized microRNA to control T leukemia cell differentiation [53,54,55,56]. When mice receive Zfra peptide followed by inoculation with cancer cells, there is a significant reduction in WWOX phosphorylation at T12, S14, Y33, and Y61 in WWOX in the spleen cells in one to two months (data not shown for T12 and S14). We believe that suppression of WWOX phosphorylation at specific sites should occur within a day or less after Zfra treatment. Since polymerized Zfra stays in the spleen for months [6], it provides a stimulatory effect for Z cell activation—that is, sustained induction of WWOX de-phosphorylation at T12, S14, Y33, and Y61 is needed for long-term Z cell activation. Indeed, antibodies against pY33-WWOX and Zfra4-10 peptide are potent in blocking cancer growth, suggesting that the Hyal-2/WWOX signaling is functioning in the anticancer response.

Based upon our knowledge, the Z cell is a novel cell lineage in the immune defense system. Autologous Z cells can be activated in vitro to exert cancer suppression in the same patient [17]. Unlike the technology of CAR-T [56], Z cell activation for cancer killing does not require prior exposure of the cells to the cancer antigens, along with tedious cloning processes. How Zfra utilizes the Hyal-2/WWOX signaling to enable Z cells to recognize and kill cancer cells is very intriguing and remains to be established.

Tumor cell heterogeneity may affect their effectiveness in colonization in a host animal. For example, we showed sharp differences in tumor sizes between two flanks from the same injections, and the occurrence rate is less than 10%. It appears that the successful colonization of cancer cells is associated with their phenotypes and the host environment. We reported that when WWOX-negative cells encounter WWOX-positive cells, the WWOX-negative cells sense the presence of WWOX-positive cells from a distance (e.g., 500 μm) and undergo retrograde migration to avoid physical contacts—that is, these cells can no longer recognize each other, even though they are from the same cell lineage [25]. Loss of WWOX in cells allows them to acquire significantly increased mobility and migration. The cells become alien to parental WWOX-positive cells [25]. In order to successfully colonize in a new WWOX-expressing organ, WWOX-negative metastatic cancer cells must partially kill a small portion of cells in the new host organ site and compromise with the host using TGF-β, so that they can successfully start docking and colonizing in the new home base [25]. Cultured cancer cells may not necessary be homogenous, as they mutate with time and the number of passages. We expect that WWOX-negative metastatic cells have more difficulty in colonizing in a new WWOX-positive host site than a WWOX-negative host microenvironment.

In summary, our new findings are: (1) Zfra4-10 reciprocally neutralizes WWOX7-21 function in cancer suppression. The neutralization is probably due to covalent binding of both peptides. (2) Zfra or WWOX peptide-mediated anticancer function in vivo is associated with its induction of endogenous WWOX interaction with its global binding partners. Covalent conjugation of Zfra with WWOX peptide leads to loss of global protein binding by endogenous WWOX and increased cancer growth. (3) Targeting membrane-associated Hyal-2 and pY33-WWOX by antibodies leads to cancer suppression in nude mice, whereas pY33-WWOX alone is not effective. Thus, directly treating mice with Zfra4-10 in the presence of pY33-WWOX may strongly block cancer growth. (4) Zfra4-10 suppresses endogenous WWOX phosphorylation at Y33 and Y61 in the spleen that leads to Z cell activation. (5) Relocation of activated Z cells to cancer lesions leads to cancer suppression. Zfra-mediated pS14-WWOX suppression in cancer lesions correlates with cancer inhibition. (6) Sonicated hyaluronan and anti-Hyal-2 antibody work in cancer suppression, suggesting that the Hyal-2/WWOX/SMAD4 signaling mediates cancer suppression. (7) We demonstrated for the first time that activated Z cells kill cancer cells in vitro. Activated Z cells have physical contacts with cancer cells. Whether conditional media from activated Z cells are cytotoxic to cancer cells remains to be established. 

Taken together, we determined the anticancer response of Zfra4-10 or WWOX7-21 peptide, which involves (1) induction of WWOX complex formation with selective target proteins in organs, and correlation with induction of cancer growth suppression; (2) de-phosphorylation of pY33- and pY61-WWOX, as it is needed for spleen Z cell activation and expansion to kill cancer cells; and (3) Zfra suppression of S14 phosphorylation of WWOX in the cancer lesions, as it is crucial for growth suppression.

## 4. Materials and Methods

### 4.1. cDNA Constructs, Site-Directed Mutagenesis, and Transient Gene Expression

A human/murine full-length Zfra cDNA and its mutant coding sequences were cloned in mammalian expression vectors such as pEGFP-C1 and pIRES2-DsRed-Express (BD Biosciences Clontech, Mountain View, CA, USA), respectively [1]. Murine *Wwox* full-length cDNA constructs in mammalian expression vectors (e.g., pEGFP-C1) were made as described [5]. The Zfra and Zfra/Wwox cDNA expression constructs were made, including Zfra-pEGFP-C1, Zfra(S6G)-pEGFP-C1, Zfra(S7G)-pEGFP-C1, Zfra(S8G)-pEGFP-C1 [2], Zfra-pIRES2-DsRed-Express, Zfra-pIRES2-Wwox-DsRed-Express, HA-Zfra(S7G)-pIRES2-Wwox-DsRed-Express, and HA-Zfra(S8G/C9W)-pIRES2-Wwox-DsRed-Express. Primers were synthesized for making Zfra(S6G) and Zfra(S7G) mutants by site-directed mutagenesis [5]: (1) S6G forward, 5′- CTATGAGCAGCAGAAGGGGGTCTTCTTGTAAATATTG-3′, (2) S6G reverse, 5′-CAATATTTACAAGAAGACCCCCTTCTGCTGCTCATAG-3′, (3) S7G forward, 5′- GAGCAGCAGAAGGTCGGGTTCTTGTAAATATTGTG-3′, and (4) S7G reverse, 5′- CACAATATTTACAAGAACCCGACCTTCTGCTGC TC-3′. Site-directed mutagenesis was performed as described [5]. Primers for cloning Wwox to pIRES2-DsRed-Express were: (1) Wwox PvuI for: 5’-CAGATATCCGATCGACCATGGCAGCTCTGCGCTAT-3’, and (2) Wwox PvuI rev: 5’-CAGATATCCGATCGGCTGGATGGACTACCCAGTC-3’. Transient gene expression in mammalian cell lines was carried out using liposome-based cell transfection reagent GeneFECTOR (Venn Nova, Pompano Beach, FL, USA), as described [5,12,13,16].

### 4.2. Antibodies, Immunohistochemistry, and Immunofluorescence Microscopy

Commercial antibodies against WWOX (N-19), WWOX (D-9), CD19 (M-20), CD27 (B-8), p53 (FL-393), p53 (DO-1), NF-2 (B-12), NFκB p65 (F-6), GFAP (2E1), CD81 (D-4), COX2 (29), C1qBP (60.11), p21 (F-5), JNK (D-2), ERK1/2 (C-9), presenilin-1 (H-70), and IL-2Rα (M-19) were from Santa Cruz Biotechnology (Santa Cruz, CA). Ki67 and CD44 antibodies were from BD Biosciences (Franklin Lakes, NJ, USA). We made specific rabbit antibodies against WWOX using synthetic WWOX286-299 peptide, and WWOX peptides with phosphorylation at Y287, pS14, and pY33, respectively [5,12,13,57,58]. Additionally, rabbit antibodies against Hyal-2 were made using synthetic peptides at amino acids #211–226 and #227–241 [12,13,58]. A phosphopeptide at pY216-Hyal-2 (amino acids #211–226) was made for immunization and antibody generation in rabbits, followed by specific antibody purification [12,13,44,50]. Immunohistochemistry and immunofluorescence microscopy were carried out using indicated tissue sections, according to our established procedures [6,7,57,58].

### 4.3. Synthetic Peptides

Zfra peptides (>95% pure) synthesized by Genemed Synthesis (San Antonio, TX) were: (1) Zfra1-31, NH-MSSRRSSSCKYCEQDFRAHTQKNAATPFLAN-COOH; (2) Zfra4–10, NH-RRSSSCK-COOH; and (3) TMR-Zfra, Zfra1-31 labeled with a red-fluorescent tetramethylrodamine (Genemed Synthesis, San Antonio, Texas, USA) [6]. These peptide stocks were made as 10 mM in degassed sterile MilliQ water. Each tube was flushed with nitrogen to prevent oxidation of serines in Zfra peptides, and the peptides were stored in a freezer at −80 °C. For tail vein injections in mice, peptides were freshly diluted at 1–4 mM in 100 μL degassed PBS and used for injection immediately to prevent over self-polymerization [6]. In addition, WWOX7-21 and WWOX7-11 peptides were made. Scrambled peptides of WWOX7-11 were also made: (1) NH-DLDGA-COOH; (2) NH-LDGDA-COOH; (3) NH-IGIDD-COOH; (4) NH-AGLEE-COOH.

### 4.4. Hyaluronan Preparations and Agarose Gel Electrophoresis for Hyaluronan

Medical grade hyaluronan was from Ginkgo-Trading Co., Ltd. (Alhambra, CA) [13]. Two mg/mL hyaluronan in sterile Milli-Q water was sonicated at 53 kHz in a water bath for 3, 6, or 8 h, and the temperature of the surrounding water could reach up to 70 °C (Soner 206H, Rocker Scientific). In UV irradiation, 2 mg/mL hyaluronan was irradiated with 3840 and 7680 mJ/cm^2^, respectively (FB-UVXL-1000, Fisher Scientific). For analyzing the molecular sizes of HA samples, 1% agarose gels were prepared in 1X TAE buffer (40 mM Tris, 82 mM acetic acid, and 8 mM EDTA, pH = 7.9). Samples containing 8 μg HA in 14 μL H_2_O were mixed with 2 μL 2M sucrose in 1X TAE buffer (containing 0.02% bromophenol blue). Electrophoresis was carried out for 2.5 h with a consistent voltage of 50 V. After the run, agarose gel was stained with 1% Alcian blue (in galactic acid, pH = 2.5) for 1 h under light protective cover, and destained overnight with 7% galactic acid.

### 4.5. Cell Lines

Cell lines from ATCC have been maintaining in our laboratory and used in the experiments, including (1) human skin basal cell carcinoma BCC [36], (2) human breast cancer MDA-MB-231 [59], (3) mouse melanoma B16F10 [6], (4) mouse breast 4T1-Luc [6], (5) human glioblastoma 13-06-MG and U87-MG [6], (6) human prostate cancer DU-145 cells [6], and (7) murine L929 fibrosarcoma cells [1,5].

### 4.6. Z Cell Activation and Purification for Killing Cancer Cells In Vitro by Time-Lapse Microscopy

For in vivo activation of Z cells, naïve mice were treated with Zfra (1 mM in 100 μL PBS) once via tail vein injection. One week later, mice were sacrificed, and spleen cells were isolated [6,7]. Spleen cells were stained with synthetic TMR (Tetramethylrhodamine)-Zfra1–31 peptide (Genemed Synthesis; excitation 550 nm, emission 573 nm). Cell sorting (FACSAria BD, Franklin Lakes, NJ, USA) was carried out to purify spleen activated Z cells. Activated Z cells (8 × 10^5^ cells) were co-cultured with mouse breast 4T1 cell monolayers in the presence of nuclear stains 4’,6-diamidino-2-phenylindole (DAPI; blue fluorescence, Calbiochem, San Diego, CA, USA) and propidium iodide (PI; blue fluorescence, Invitrogen, Carlsbad, CA, USA) for imaging by time-lapse microscopy using an inverted Olympus IX81 microscope [9,10,13,16,25]. The extent of DAPI uptake by live cells indicates increases in nuclear membrane permeability and PI uptake for cell death [9,16].

### 4.7. Experimental Models: Animal Models and Cancer Cell Growth In Vivo

Use of animals in experiments was approved by the Intramural Animal Use and Care Committee (IACUC) of the National Cheng Kung University (NCKU). Furthermore, we followed the US NIH guidelines in animal experiments. Six- to eight-week-old male BALB/c mice, NOD-SCID (NOD.CB17-*Prkdc^scid^*/NCrCrl) mice (Laboratory Animal Center, NCKU), or nude (BALB/cAnN.Cg-*Foxn1^nu^*/CrlNarl) mice (National Laboratory Animal Center, Taiwan) were used. Mice were inoculated with B16F10, BCC, 4T1, MDA-MB-231, 13-06-MG, U87-MG, or other indicated cells in both left and right flanks and then received Zfra4–10 peptide (1 mM in 100 μL PBS) or PBS via tail vein injections one week later. Alternatively, tumor cells were allowed to grow up to 200 mm^3^ prior to peptide treatment. In addition, mice received intravenous injections of Zfra peptide, followed by inoculation with B16F10 in both flanks. Tumor volumes were measured twice per week and calculated: *D* x (*d*)^2^/2, where *D* and *d* are the major and minor diameters, respectively. Where indicated, tissue sections of spleen, lung, liver, and tumor lesions were prepared for immunohistochemistry using specific antibodies [6,7].

### 4.8. Organ Lysates and Co-Immunoprecipitation

5 to 10 organs were harvested after mice were sacrificed. These organs were immersed in 1 to 2 mL PBS containing 5 mM phenylmethylsulfonyl fluoride (PMSF) and 1 mM sodium orthovanadate (Na3VO4, pH 7.4) and stored in the freezer at −80 °C. Prior to co-immunoprecipitation [5,10,11,12,13], organs were sonicated and centrifuged at 14,000 rpm for 20 min. The organ lysates were harvested and the protein concentrations were measured (BioRad Protein Assay, BioRad). 500 μg of organ lysates were precleared with 15 μL protein G agarose beads (Invitrogen) for 1 h, followed by adding a specific antibody (3 μg) and 23 μL protein agarose G beads for incubating in an end-over-end shaker for 6 to 18 h. Following washing with PBS and centrifugation for 4 times, the beads were added with an aliquot of reducing sample buffer and antibody-bound proteins were released by heating the beads at 90 °C for 10 min. Finally, SDS-PAGE, electrotransfer to nitrocellulose membranes, and Western blotting were carried out [5,10,11,12,13].

### 4.9. Quantification and Statistical Analysis

In immunohistochemistry, 5–6 tissue sections were stained with a specific antibody and a representative set of data set is shown. A representative data from the kinetics of tumor cell growth in tumor-bearing mice is shown from 2–6 experiments, as specified. All animal experiments were repeated 2–3 times. For statistical analysis, the non-paired Student’s *t* test was used to examine the differences among controls and tested groups, as analyzed using Microsoft Excel software. Data were expressed as mean ± standard deviation, where *p* < 0.05 was considered as statistically significant.

### 4.10. Ethics Approval in Animal Use

All experiments involved in animal use have been approved by the Institutional Animal Care and Use Committee (IACUC) of the National Cheng Kung University College of Medicine (Approval numbers 105064, 105070, 106064, 107027, 107080, 107296, 108041, 108153, and 110001).

## 5. Conclusions

Both synthetic Zfra4-10 and WWOX7-21 peptides strongly suppress cancer growth in vivo. Supporting evidence revealed that activation of the Hyal-2/WWOX/SMAD4 signaling by the peptides is responsible for induction of cytotoxic Z cell activation. Notably, Zfra4-10 or WWOX7-21 peptide induced the binding of endogenous WWOX with ERK, C1qBP, NF-κB, Iba1, p21, CD133, JNK1, COX2, Oct4, and GFAP in the spleen, brain, and/or lung, which correlates with cancer suppression. Loss of this binding allows cancer growth, suggesting a novel mechanism of WWOX-mediated cancer suppression in vivo. Finally, we developed Zfra4-10 and WWOX7-21 peptides, sonicated hyaluronan HAson, and Hyal-2 and pY33-WWOX antibodies as therapeutic drugs for cancer treatment.

## 6. Patents

1. Nan-Shan Chang, and Wan-Pei Su. Modified hyaluronan and uses thereof in cancer treatment. US Patent number 9375447; 14 March 2013.

2. Nan-Shan Chang, Chen-Yu Lu, Wan-Pei Su, Yu-An Chen, Wan-Jen Wang. Z cells activated by zinc finger-like protein and uses thereof in cancer treatment. US Patent number 9546354; 17 January 2017.

## Supplementary Materials

The following are available online at https://www.mdpi.com/2072-6694/12/8/2189/s1, Supplemental Figures: Figure S1: Functional neutralization between Zfra and WWOX peptides in controlling cancer growth; Figure S2: Spleen Z cell isolation by cell sorting; Figure S3: Z cell clonal expansion in the spleen; Figure S4: CD19+ or CD27+ B cells do not contribute to Zfra-mediated cancer growth suppression; Figure S5: Activated Z cells attack and kill 4T1 cells; Figure S6: 4T1 cell death via whole cell explosion or popping out. Figure S7. Functional neutralization between Zfra and WWOX in controlling cancer growth. (Data shown in Figure 1I; see main text). Figure S8. Zfra or WWOX peptide upregulates the binding of endogenous WWOX with specific proteins in organs of mice, which correlates with cancer growth suppression. (Data shown in Figure 2B; see main text). Figure S9. Zfra or WWOX peptide upregulates the binding of endogenous WWOX with specific proteins in organs of mice, which correlates with cancer growth suppression. (Data shown in Figure 2C; see main text). Figure S10. Zfra or WWOX peptide upregulates the binding of endogenous WWOX with specific proteins in organs of mice, which correlates with cancer growth suppression. (Data shown in Figure 2D,E; see main text). Supplemental Videos: Videos S1–S3: Zfra-activated spleen cells attacked breast 4T1 cells; Videos S4–S6. Control spleen cells did not attack breast 4T1 cells; Video S7. Activated Z cells induced death of 4T1 cells; Videos S8–S10. Breast 4T1 cells died by whole cell explosion; Videos S11–S12: WWOX7-21-activated Z cells killed 4T1 cell spheres; Videos S13–S14: Scrambled WWOX7-11-activated Z cells did not kill 4T1 cell spheres. Figures with digital data for Western blots: Figure S11. Functional neutralization between Zfra and WWOX in controlling cancer growth. Data for 1I and 1J; Figure S12. Zfra or WWOX peptide upregulates the binding of endogenous WWOX with specific proteins in organs of mice, which correlates with cancer growth suppression. Data from Figure 2B to Figure 2E.
